# Beyond the bubble: reflections on a career in academic medicine with an obligation to look beyond

**DOI:** 10.1002/acm2.70308

**Published:** 2025-10-22

**Authors:** Timothy D. Solberg

**Affiliations:** ^1^ Department of Radiation Oncology University of Washington Seattle Washington USA

**Keywords:** governance, health policy, professional privilege, public trust

We live in an increasingly polarized world, one where communication itself feels fractured and mutual understanding is elusive. At times, the rhetoric can drown out honest dialogue—even among friends and colleagues who share common goals. Against this backdrop, it is tempting for academic medicine to become defensive and turn inward. But retreating further into our own bubble of prestige, privilege, and self‐reinforcing narrative would obviously be a mistake. Having spent my career in academia, I find myself asking not just how we see ourselves, but how others see us. That perspective—often absent from our self‐assessments—matters now more than ever.

Academic medicine positions itself as a bastion of discovery, equity, and public service, yet too often it operates in ways that appear insulated, entitled, and unaccountable. Faculty enjoy privileges that most sectors would find enviable: generous startup packages, semi‐autonomous work environments, protected professional time, travel support, sabbaticals, and the prestige of self‐reinforcing titles and promotions. Hiring and advancement frequently occur within narrow networks, perpetuating insularity rather than welcoming fresh ideas or accountability.

These advantages come at a cost often invisible from within the system. While academics may view research support, sabbatical leaves, and travel expenses as earned benefits, to the public, they can appear as taxpayer‐supported subsidies. The federal research enterprise—principally through NIH, DOE, and NSF—represents a direct transfer of taxpayer dollars into universities. Like agricultural, energy, education, housing, and other subsidies, these funds come from a finite pool. Every dollar that sustains a laboratory, supports protected time, underwrites conference travel, or funds a sabbatical is a dollar not spent on alternative energy, affordable housing, or food security. It is a dollar not spent on repairing bridges, modernizing water systems, expanding public health clinics, or supporting education and childcare. Ignoring the finite nature of these resources only reinforces the perception of entitlement.

Too often our leadership responds to these realities not with candor but with clichés. We invoke our “north star” or “true north” as though such metaphors could substitute for meaningful reform. They cannot. Empty slogans do not fix inefficiencies or inequities, nor do they rebuild public trust. Likewise, constant refrains that we are working too hard or need more resources may ring true within our walls but sound tone‐deaf outside them. Simply asking for more people or larger budgets is not a strategy—it is avoidance.

The real work requires less rhetoric and more resolve. Leaders must roll up their sleeves, listen to those who deliver care and teach students, and confront the realities of how our institutions actually function. It means mapping workflows honestly, identifying inefficiencies, and acknowledging biases that distort promotions, research priorities, and patient care. It means managing down, not just up—supporting those on the ground rather than protecting hierarchies. And it means actualizing health equity, not as a lofty aspiration but as a measurable outcome embedded in hiring, training, and care delivery.

History offers cautionary parallels. The U.S. auto industry leaned on subsidies to sustain inefficiencies, only to face collapse when the public lost patience. Newspapers believed they were indispensable until the digital revolution hollowed them out. Political parties have closed ranks to protect privilege, only to find themselves abandoned by the very voters they assumed were loyal. Higher education as a whole is facing similar backlash today. Each ignored warning signs, each assumed indispensability—and each discovered that public trust is finite. Academic medicine risks the same fate if it clings to its bubble, ignoring inefficiencies and defending entitlements rather than confronting them.

The warning signs are already here. Indirect costs on NIH grants frequently exceed 50%, sending more dollars to central administration than to science. “Protected time” translates into reduced clinical effort, leaving community clinicians to absorb the workload. Effort targets are quietly adjusted downward to accommodate “academic activity.” Travel and conference attendance are routinely subsidized while frontline clinicians struggle to meet even their required continuing education. Faculty enjoy sabbaticals and paid volunteer work while nurses and residents face escalating patient loads. Academic promotions reward publications and grant dollars, while the daily realities of patient care are undervalued. These practices may feel justified inside our bubble, but outside, they look like a system more committed to protecting itself than serving the public.

Professional societies are not exempt. Too often, they act as guilds, defending reimbursement rates and amplifying entitlements while collecting membership dues, conference fees, and publishing revenues that are, in turn, underwritten by academia and government. Leadership positions are frequently circulated within the same insular networks, reinforcing privilege rather than broadening opportunity. Societies celebrate scientific and political advances, but rarely confront institutional inefficiency or the misalignment between commercial and professional interests and public need. They lobby vigorously for incremental gains in reimbursement yet remain silent on the rising cost of care or the trade‐offs in research spending. Publishing adds another layer of complexity: when societies rely on journal revenues to sustain operations, the line between member service and public service blurs. True editorial independence—ensuring that publications exist to advance science and patient care rather than to advance individual promotion or subsidize professional perks—is essential to credibility. If societies in academic medicine are to remain credible, they must not only defend science, prioritize patient care, and safeguard editorial independence, but also be mindful of their civic responsibilities to ensure the responsible use of public funds. Encouragingly, some societies are beginning to pivot—investing in community‐facing prevention and health equity, addressing drug pricing and access to care, and advancing global initiatives on AI ethics and open science.

This is not about demeaning academic medicine. For me, it is about learning to find humility again. It is about understanding how others see us, especially in a political climate where academic institutions are increasingly painted as elitist, inefficient, and out of touch. In a polarized environment, dismissing these perceptions as mere rhetoric is perilous. Perceptions shape trust as much as realities do, and once trust is lost, it is extraordinarily difficult to regain. History makes this clear: banks after the 2008 financial crisis, automakers after the 2009 bailouts, newspapers in the digital age, and political parties that mistook loyalty for permanence all discovered that public trust, once eroded, is rarely restored. These narratives drive policy decisions and budget priorities. They influence whether the subsidies that sustain us are protected, redirected, or withdrawn altogether. Ignoring them does not make them go away—it only deepens the perception that we are unwilling or unable to change. Trust in healthcare organizations has been declining for decades, a trend documented in national surveys showing erosion of confidence in hospitals, medical leaders, and public health agencies.^1^ The COVID‐19 pandemic accelerated this decline, amplifying public skepticism through political polarization, inconsistent communication, and visible strains on health systems.^2^ What had been a slow drift of eroding trust is becoming a rupture—leaving many institutions struggling to reestablish credibility and financial accountability at precisely the moment when public reliance on them is most critical.

The choice before us is stark. We can remain in our bubble—clinging to slogans, defending privilege and entitlements, and convincing ourselves that public support is guaranteed simply because we are educated and presume to know best. But that path leads only to irrelevance and reform imposed from the outside. Or we can step beyond the bubble—acknowledge our dependence on public trust, confront our entitlements and inefficiencies honestly, and recommit ourselves to the missions that matter: Caring for patients, training the next generation, and advancing discoveries that truly improve health.

This is not an abstract exercise; it is a lived experience for me. I have spent my career inside this bubble, benefiting from its privileges and witnessing the blind spots it creates. One lesson is clear: Bubbles do not last forever. As Warren Buffett observed, “Trust is like the air we breathe—when it's present, nobody really notices; when it's absent, everybody notices.” For academic medicine to endure, it must reclaim humility over entitlement, action over slogans, and transparency and accountability over tradition. Only then can we honor the public trust on which our survival depends.

## AUTHOR CONTRIBUTIONS

Timothy Solberg was responsible for conceptualization, investigation, writing, reviewing, and editing.

## CONFLICT OF INTEREST STATEMENT

The author reports no relevant conflicts of interest related to this work. The author is a part‐time employee of the University of Washington, is a managing partner of Global Radiosurgery Services, and is the CEO and co‐founder of Foretell Medical. The author is a deputy editor‐in‐chief for the JACMP and is compensated by the AAPM for this service. ChatGPT 5.0 was used in the proofreading of this editorial.

## References

[1] Blendon RJ , Benson JM . Trust in medicine, the health system & public health. Dædalus J Am Acad Arts Sci. 2022;151(4):67‐82. doi:10.1162/daed_a_01944

[2] Perlis RH , et al. Trust in physicians and hospitals during the covid‐19 pandemic in a 50‐state survey of us adults. JAMA Netw Open. 2024(7):e2424984. doi:10.1001/jamanetworkopen.2024.24984 39083270 PMC11292455

